# Decreased Dengue Replication and an Increased Anti-viral Humoral Response with the use of Combined Toll-Like Receptor 3 and 7/8 Agonists in Macaques

**DOI:** 10.1371/journal.pone.0019323

**Published:** 2011-04-29

**Authors:** Carlos A. Sariol, Melween I. Martínez, Francheska Rivera, Idia Vanessa Rodríguez, Petraleigh Pantoja, Kristina Abel, Teresa Arana, Luis Giavedoni, Vida Hodara, Laura J. White, Yesseinia I. Angleró, Luis J. Montaner, Edmundo N. Kraiselburd

**Affiliations:** 1 Unit of Comparative Medicine, Caribbean Primate Research Center, University of Puerto Rico-Medical Sciences Campus, San Juan, Puerto Rico, United States of America; 2 Department of Microbiology and Medical Zoology, University of Puerto Rico-Medical Sciences Campus, San Juan, Puerto Rico, United States of America; 3 Department of Internal Medicine, University of Puerto Rico-Medical Sciences Campus, San Juan, Puerto Rico, United States of America; 4 Department of Microbiology and Immunology, School of Medicine, University of North Carolina at Chapel Hill, Chapel Hill, North Carolina, United States of America; 5 Departments of Virology and Immunology, Texas Biomedical Research Institute, San Antonio, Texas, United States of America; 6 The Carolina Vaccine Institute, University of North Carolina, Chapel Hill, North Carolina, United States of America; 7 The Wistar Institute, Philadelphia, Pennsylvania, United States of America; University of California, San Francisco, United States of America

## Abstract

**Background:**

Pathogenic versus protective outcomes to Dengue virus (DENV) infection are associated with innate immune function. This study aimed to determine the role of increased TLR3- and TLR7/8-mediated innate signaling after Dengue infection of rhesus macaques *in vivo* to evaluate its impact on disease and anti-DENV immune responses.

**Methodology/Principal Findings:**

TLR3 and TLR7/8 agonists (emulsified in Montanide) were administered subcutaneously to rhesus macaques at 48 hours and 7 days after DENV infection. The Frequency and activation of myeloid dendritic cells, plasmacytoid dendritic cells, and B cells were measured by flow cytometry while the serum levels of 14 different cytokines and chemokines were quantified. Adaptive immune responses were measured by DENV-specific antibody subtype measurements. Results showed that the combined TLR agonists reduced viral replication and induced the development of a proinflammatory reaction, otherwise absent in Dengue infection alone, without any clear signs of exacerbated disease. Specifically, the TLR-induced response was characterized by activation changes in mDC subsets concurrent with higher serum levels of CXCL-10 and IL-1Ra. TLR stimulation also induced higher titers of anti-DENV antibodies and acted to increase the IgG2/IgG1 ratio of anti-DENV to favor the subtype associated with DENV control. We also observed an effect of DENV-mediated suppression of mDC activation consistent with prior *in vitro* studies.

**Conclusions/Significance:**

These data show that concurrent TLR3/7/8 activation of the innate immune response after DENV infection *in vivo* acts to increase antiviral mechanisms via increased inflammatory and humoral responses in rhesus macaques, resulting in decreased viremia and melioration of the infection. These findings underscore an *in vivo* protective rather than a pathogenic role for combined TLR3/7/8-mediated activation in Dengue infection of rhesus macaques. Our study provides definitive proof-of-concept into the mechanism by which DENV evades immune recognition and activation *in vivo*.

## Introduction

Dengue is the most important arboviral disease, with 50–100 million cases of Dengue Fever (DF) and 500,000 cases of Dengue Hemorrhagic Fever (DHF)/Dengue Shock Syndrome (DSS) each year [1]. No specific, primary or secondary prevention treatments for Dengue are available. The disease is transmitted by either of four dengue virus (DENV) serotypes (DENV-1, -2, -3 and -4). The physiopathology of Dengue virus infection has been extensively studied, and the disproportionate induction of pro-inflammatory cytokines after infection is associated with capillary leakage and hemorrhagic manifestations [2], [3]. The importance of the adaptive immune responses in the outcome of DENV infection is well established [4], [5]. However, more recently a key role in determining the course of DENV infection has been attributed to the innate immune response, particularly to the pattern-recognition receptors (PRRs) such as Toll-like receptors 7/8 (TLRs) [6], [7], [8]; the retinoic acid-inducible gene/melanoma differentiation gene 5 (RIG-I/MDA5) [9], [10]; and the interaction between DENV and the interferon signaling pathway [11], [12], [13]. The DENV unfragmented, single-stranded RNA genome is recognized by TLR7 [6], [8], [14], whereas double-stranded RNA (dsRNA), an intermediary product during viral replication, can be recognized by TLR3 [14], [15]. The role of the innate immune response, particularly of dendritic cells (DC) at early stages after DENV infection, has become a focus of great interest [16], [17], [18]. Studies *in vitro* show that DENV induces DC activation and maturation [19], [20]; however, the profile of activation/maturation differs between *in vitro*-infected and non-infected bystander DC [14], [20], [21], [22]. In particular, TLR7 is implicated in the functional response of plasmacytoid DC (pDC) to DENV [8], [14] and plays an essential role in the activation of the adaptive immune response [23], [24]. However, no study to date has explored the role of innate activation in *in vivo* models of Dengue infection.

The rhesus macaque is an established non-human primate model for the study of the innate immune response to different viruses, including Dengue [25], [26], [27], [28], [29]. Monkeys pre-treated with a TLR3 agonist did not die after they were challenged with a virulent strain of yellow fever (YF). Moreover, they developed neutralizing antibodies against YF [30]. In another study, fewer animals treated with TLR3 agonist developed viremia or the viremia was delayed after they were challenged with Venezuelan Equine Encephalomyelitis (VEE) virus [31], consistent with an antiviral role for concurrent TLR activation.

More recently, it was shown that local immunization at the vaginal mucosa with a TLR7 agonist induced a strong innate immune response and activation of local CD4^+^ T cells in rhesus macaques [29]. When TLR7/8 and 9 agonists, diluted in phosphate-buffered saline (PBS) or emulsified in Montanide, an oil-based adjuvant, were administered subcutaneously (s.c.), the magnitude and quality of the humoral and T helper (TH) 1 cellular immune response to human immunodeficiency virus HIV Gag protein was boosted [32], [33]. Subcutaneous administration of different TLR3 agonists in combination with an aqueous solution of keyhole limpet hemocyanin (KLH) induced DC activation and the stimulation of TH1 and humoral immune responses to human papillomavirus [34]. Despite the well-established role of combined TLR 3 or 7/8 effects in the activation of immune responses against many viruses, little is known about their combined role in relationship to Dengue infections *in vivo*. Specifically, it has remained unknown if concurrent inflammation by TLR activation alone after DENV infection may exacerbate symptoms and contribute to capillary leakage and hemorrhagic manifestations of infection or whether these clinical events are indicative of a response involving both innate and adaptive components.

Here, we demonstrate that combined TLR3 and 7/8 stimulation after an established DENV infection results in the activation of myeloid DC (mDC) and in the quantitative and qualitative modification of the adaptive immune response without exacerbating the disease, supporting a protective role for concurrent multi-TLR-mediated activation as in the studies noted above. We also show that DENV is able to counteract the mDC activation and CXCL-10 production induced by the TLR agonists. Although TLR agonists alone were not sufficient to induce a significant activation of B cells, in combination with DENV they induced B-cell activation and altered the switch of IgG classes, indicating that reductions in viral replication after TLR agonists administration may be associated with more potent anti-DENV responses.

## Materials and Methods

### Animals, virus, and procedures

Fourteen Indian rhesus macaques were stratified into comparable groups based on age, weight and sex. Groups 1 (n = 3; DENV) and 2 (n = 4; DENV/TLR) were infected s.c in the deltoid area with 1 ml of 1×10^4^ plaque forming units (pfu) of a low-passage Western Pacific 74 DV1 strain (Dr. L. Markoff, Walter Reed Army Medical Center).

In addition, Group 2 animals received two doses of TLR-7/8 agonist CL097M-012 (1 mg in 200 ml/animal) and TLR3 agonist poly (I:C) (InvivoGen, San Diego, CA) (2 mg/ml/animal) in a 70% (v/v) emulsion of Montanide ISA 51 (InvivoGen, San Diego, CA) on days 2 and 7 post infection. The TLR agonists were administered s.c. in two separate regions in the interscapular area. Based on previous studies, we decided to use a fixed standardized dose of 2 mg/animal and 1/mg per animal of poly (I:C) and CL097M-012, respectively, rather than to adjust the dose based on body weight, as this dose resulted in the stimulation of innate and/or adaptive immune responses in rhesus monkeys [30], [31], [33], [35]. Control group 3 (n = 4; TLR) received only the TLR agonists as described for Group 2. Control group 4 (n = 3; Control) received 1 ml (s.c. deltoid area) of supernatant from mock-infected Vero cells.

All procedures were reviewed and approved by the Institute's Animal Care and Use Committee at Medical Sciences Campus, University of Puerto Rico (IACUC-UPR-MSC), and performed in a facility accredited by the Association for Assessment and Accreditation of Laboratory Animal Care (AAALAC). Animal Welfare Assurance Number: A3421, Protocol number: 7890108. In addition, steps were taken to ameliorate suffering in accordance with the recommendations of the Weatherall report, “The Use of Non-human Primates in Research”. For instance, to ameliorate the suffering of the animals, all procedures were conducted under anesthesia by using ketamine 10–20 mg/kg IM as approved by IACUC. Anesthesia was delivered in the caudal thigh using a 23 gauge sterile syringe needle. Continued monitoring was provided by a trained Veterinarian at the Animal Research Center. During the time of the protocol, animals were under the Environmental Enrichment program of the facility, also approved by IACUC.

### PBMC Separation

Sera and peripheral blood mononuclear cells (PBMC) were collected on days 1, 4, 10 and 30 post infection. Cells were preserved in liquid nitrogen for FACS analysis.

### RNA extraction, viremia and DENV NS1 protein detection

RNA was extracted from 400 ml of serum using an RNeasy Mini Kit (Qiagen, Valencia, CA), and eluted in 30 ml of RNase-free H_2_O. The quality and quantity of RNA were determined using Agilent Bioanalyzer RNA Nanochip technology.

RNA extracted from sequentially collected serum samples in the first 10 days after infection was used to quantify viremia by Real-Time RT-PCR (qRT-PCR) using an iQ5 machine (BioRad Laboratories, Hercules, CA) following previously published procedures [36]. Results were confirmed in plasma samples by using the Platelia Dengue NS1 Ag Kit (Bio-Rad, Marnes-la-Coquette, France) following the manufacturer's instructions.

### Flow cytometric analysis

PBMC (10^6^) collected on days 1, 4 and 10 after infection were stained with anti-human antibodies cross-reactive with rhesus macaques. Cells were also incubated with isotype-matched immunoglobulin as a negative control. These antibodies included a cocktail of linage-specific antibodies [fluorescein isothiocyanate (FITC)–conjugated CD3 (clone SP34), CD16 (clone 3G8), CD20 (clone 2H7), CD14 (clone M5E2)] and antibodies used for DC characterization [peridinin chlorophyll protein (PerCP Cy5.5)-conjugated CD86 (clone IT2.2), phycoerythrin (PE)-conjugated CD40 (clone 5C3), Alexa Fluor ®700-conjugated HLA-DR (clone L243), and allophycocyanin (APC)-conjugated CD123 (clone 7G3) or CD11c (clone S-HCL-3) for pDC and mDC, respectively. PBMC collected on day 10 and 30 were stained with specific markers for B cells [FITC-conjugated CD20 (clone 2H700), APC-conjugated CD3 (clone SPE34-2)]. For activation of these cells a PE-conjugated anti-CD69 (clone FN50) was used. After 30 min of incubation at 4°C in the dark, the PBMC were washed with PBS and 300 µL of 1% formaldehyde was added to fix the samples. The samples were stored at 4°C in the dark and analyzed within 6 hrs on a BD FACSAria (BD Biosciences). Data were analyzed using Summit Software (version 3.1). The frequencies of activated cells are reported as a percentage of the specific cell population. Antibodies against CD86 and HLA-DR were purchased from Biolegend (San Diego, CA) and anti-CD69 was obtained from Dako (Carpentaria, CA). All other antibodies were purchased from BD Pharmingen (San Jose, CA).

### Antibody measurements

Prior to study entry, all animals were tested for the presence of both anti-Dengue IgM ad IgG. The IgM antibody-capture enzyme-linked immunosorbent assay (MAC-ELISA) and the Dengue-IgG antibody tests were performed using a quantitative in-house ELISA procedure described elsewhere [37].

Neutralizing antibodies were determined in samples collected 30 days after the infection, and antibody isotyping was performed on serum samples from days 10, 15 and 30. Neutralizing antibodies were determined by the FACS neutralization test (FNT) as previously described [38]. An in-house indirect enzymatic immune assay system was developed for the determination of DENV-specific IgG classes. A DENV-2 antigen with known cross-reactivity to all DENV serotypes was used (Fitzgerald Industries International, Acton, MA). Mouse monoclonal anti-human IgG1 and IgG2 antibodies (Sigma) were used for subclass classification. The ratios of DENV-specific IgG subclass antibodies were calculated with the following formula: ratio  =  [(OD of sample - OD of blank) – (average of negative control)]/(OD IgG1/OD IgG2) and vice versa, where OD is the optical density of each sample.

### Multiplex cytokine/chemokine detection

Serum samples collected on days 1, 4, 10 and 15 after infection were used to detect the presence of 14 nonhuman primate chemokines and cytokines [CXCL-10 (IP-10), MIP-1β, TNF-α, G-CSF, IFN-α, IFN-γ, IL-12 (p40), IL-17, IL-18, IL-1β, IL-1Ra, IL-4, IL-5, IL-6], using the Luminex100 system as previously described [37]. The raw data (mean fluorescence intensity) from all the bead combinations tested were analyzed with Master-Plex QT quantification software (MiraiBio Inc., Alameda, CA) to obtain concentration values in pg/ml. Baseline values were determined with samples collected from animals in the control group at each time point.

### Statistical analysis

Values were expressed as mean ± standard error of the mean (SEM). Significant differences were determined by Student's t-test or the Mann Whitney U-test. Two-way analysis of variance (ANOVA) tests followed by the Bonferroni multiple comparison test correction and the Kruskal-Wallis test were used. When applied, further differences between groups were tested by un-paired t-tests. Analyses were performed using Prism software (version 5.0c; GraphPad Software Inc., San Diego, CA). A *P* value of <0.05 was considered to represent a significant difference with (*) p<0.05, (**) p<0.01, and (***) p<0.001.

## Results

### Effect of TLR agonists on the outcome of DENV-1 infection

The effectiveness of poly (I:C) and CL097M-012 as agonists for TLR-3 and TLR-7/8, respectively, to modulate immune responses in rhesus macaques was previously established *in vivo* [31], [32], [33], including in studies of YF, another member of the Flaviviridae family [30]. Based on these data, and to facilitate comparison with previous studies, we used the same doses in the current studies.

**Figure 1:**
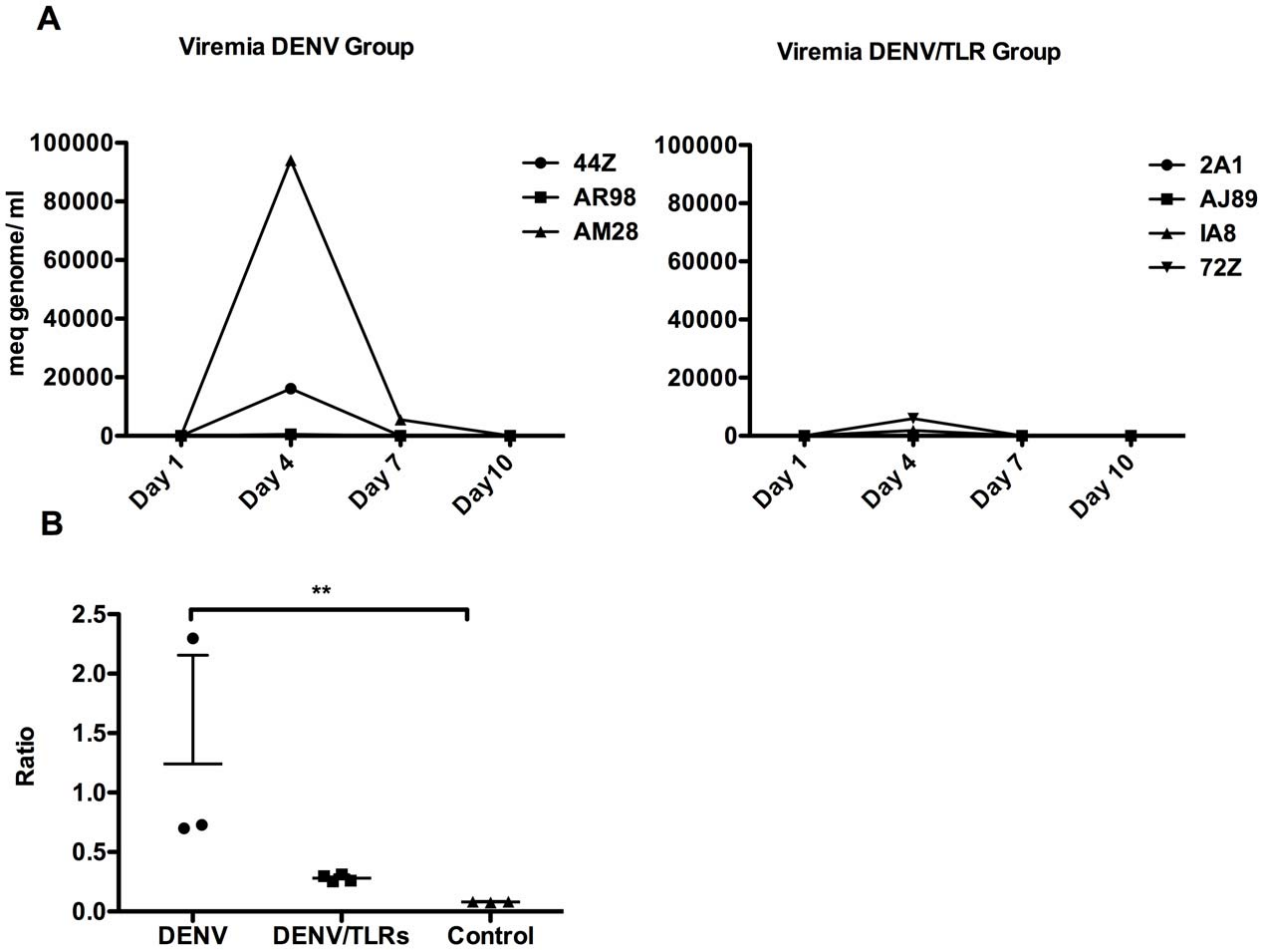
Toll-like receptor agonists abrogate viremia. A: Left panel, a peak of viremia was detected at day 4 after DENV infection in two of three animals (44Z and AM28). However, this peak was completely absent in the four animals of the DENV/TLR group at 48 hours after the TLR3 and 7/8 agonists [poly (I:C) and CL097M-012, respectively] were administered (right panel). Results are expressed in milliequivalent genomes per milliliter. B: Plasma levels of DENV protein NS1 were not significantly different between the DENV group and the treated group (DENV/TLR). However, NS1 levels were significantly higher in the DENV group compared to the Control group (p<0.018), whereas they were not significantly different between the DENV/LTR (despite the Dengue infection) and the Control groups. This confirmatory assay corroborates the role of TLRs in controlling DENV replication. The results reflect the ratio of the sample O.D./Control O.D. according to the manufacturer's instructions.

Viremia was detected by qRT-PCR in 2 of 3 animals infected with DENV, with peak viremia occurring on day 4 after infection (Fig. 1A, left panel). Interestingly, the viremia was abrogated in 3 of the 4 DENV/TLR animals (75%) and remained at the minimal level of detection in the other treated animal (25%) (Fig. 1A, right panel). The results of the viral load assay correlate with the levels of DENV NS1 protein in plasma (Fig. 1B). Two animals in the DENV group were equivocal and one was highly positive for the NS1 viral protein 4 days after infection. The index value of this group was significantly higher when compared to the Control group (*p*<*0.018*), whereas the 4 DENV/TLR animals remained negative without a significant difference compared to the Control group despite being infected with DENV under similar conditions to animals in the DENV group. Taken together, administration of combined TLR agonists during the acute phase of DENV infection was associated with control of viral replication.

### Effect of TLR agonists on the innate immune response in vivo

**Figure 2:**
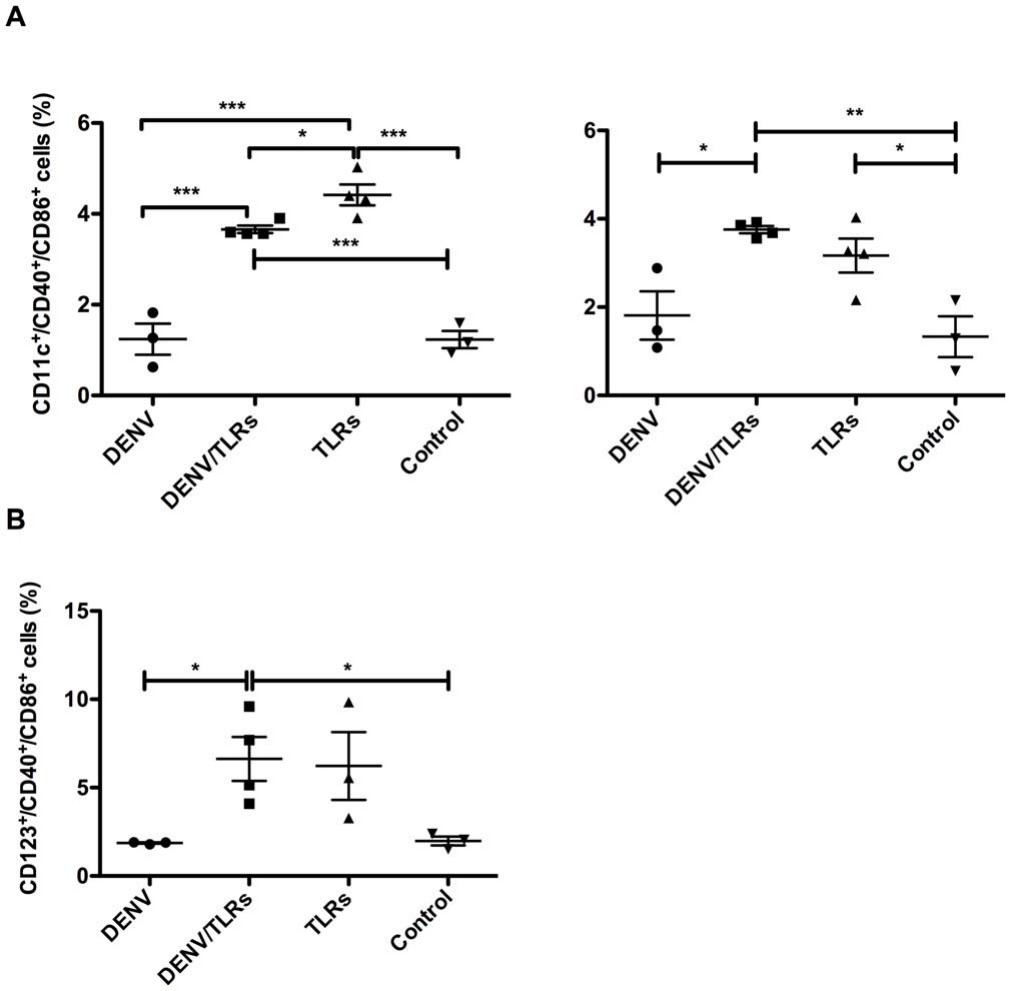
Dengue virus inhibits TLR-induced activation of mDC. A: Left panel, 48 hours after the first TLR stimulation, mDC were activated in the groups receiving TLRs but not in the group receiving only the virus. Coincident with the peak of viremia at day 4, an inhibitory effect of DENV on the mDC subset was evidenced by the frequency of activated cells in the DENV group, which was significantly lower compared to the DENV/TLR and TLR groups. Right panel, 10 days after the infection (72 hours after second TLR stimulation) the mDC subset was still significantly activated in both groups that received TLRs when compared to the Control group. However, the inhibitory effect of DENV was absent as there were no significant differences between the DENV and DENV/TLR groups, supporting the role of DENV replication in the inhibitory effect on mDC activation on day 4. B: Ten days after infection and 72 hours after the secondary TLR stimulation the frequency of activated pDC was significantly higher in the DENV/TLR and TLR groups compared to the DENV and Control groups. However, an inhibitory effect of DENV on this subset of DC was not observed. Asterisks denote significant differences, (*) p <0.05, (**) p <0.01, and (***) p<0.00.

To establish a potential contribution of TLR stimulation on effector cells of the innate immune response after DENV infection *in vivo*, we studied the activation of pDC and mDC in peripheral blood. We examined: (i) day 1 post infection (prior to TLR stimulation), and (ii) days 4 and 10 post infection corresponding to 48 and 72 hours after the first and second TLR administrations, respectively. On day 1 post infection, no activation of DC compared to day 0 was detected (data not shown). However, on day 4 post infection, the frequencies of the activation markers CD40^+^ and CD86^+^ on mDC were significantly higher in the TLR group (4.418% ±0.231% SEM) compared to all other groups (DENV/TLR: 3.66% ±0.080% SEM, *p*<*0.021*; DENV: 0.950% ±0.320% SEM, *p*<*0.001*; Control: 1.233% ±0.190% SEM, *p*<*0.001*). Animals in the DENV/TLR group also showed higher frequencies of activated mDC when compared to the DENV-infected and Control groups (Fig. 2A, left panel). No statistically significant differences in the percentage of activated pDC (Lin^−^/HLA-DR^+^/CD123^+^/CD40^+^/CD86^+^) were detected on day 4 post infection between groups (data not shown).

However, on day 10 post infection (72 hours after the second administration of TLR agonists), both mDC (Fig. 2A, right panel) and pDC (Fig. 2B) showed a higher frequency of activation in the DENV/TLR group when compared to the DENV or Control groups (for mDC: 3.755% ±0.082% SEM vs. DENV 1.810% ±0.546% *p*<*0.008*; and vs. Control 1.330% ±0.462% SEM *p*<*0.001*; for pDC 6.628% ±1.244% SEM vs. DENV 1.870% ±0.035% SEM *p*<*0.023*; and vs. Control 1.980% ±0.250% SEM *p*<*0.026*). Animals in the combined TLR group alone had statistically higher frequencies of activated mDC (Fig. 2A, right panel), but not of pDC (Fig. 2B), when compared to the Control group (3.168% ±0.384% SEM vs. 1.838% ±0.596% *p*<*0.027*). At this later time point, no statistical differences were observed between the DENV/TLR and TLR groups, reinforcing the anti-inflammatory response by DENV in the results obtained during the viremia peak observed on day 4 (Fig. 2A, left panel).

### Induction of pro-inflammatory cytokines and chemokines

**Figure 3:**
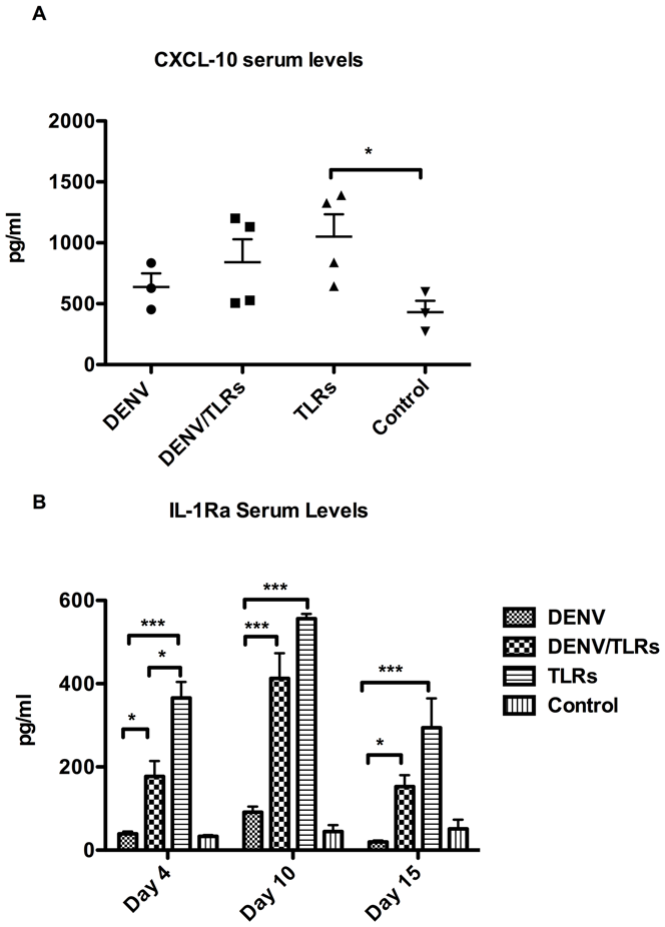
Serum levels of CXCL-10 and IL-1Ra are increased significantly after TLR stimulation and are inhibited by DENV. A: Coincident with the higher level of mDC activation, serum levels of CXCL-10 increased significantly only in the TLR group when compared to the Control group. Higher levels, albeit not significant, were also found in the DENV/TLR group. The lowest levels among the experimental groups were found in animals of the DENV group. This trend in the serum levels of CXCL-10 among different groups confirms a stimulatory effect of the TLR agonist but at the same time suggests an inhibitory role of Dengue virus in the secretion of this cytokine. B: Serum levels of IL-1Ra peaked 48 hours after the first TLR stimulation. An inhibitory effect of DENV at this time point (also coincident with the viremia peak) is supported by significantly higher values of this cytokine in the TLR group when compared to the DENV and DENV/TLR groups. Both groups, DENV/TLR and TLR, showed significantly higher serum levels of this cytokine when compared to the DENV and Control groups. Ten days after the infection (72 hours after second TLR stimulation), IL1-Ra values were still increased in both groups receiving TLR agonists. Significantly higher levels were present up to 15 days after the infection (8 days after the last TLR stimulation). However, at the later time point, there was no significant difference between the DENV/TLR and TLR groups. Asterisks denote significant differences, (*) p <0.05, (**) p<0.01, and (***) p<0.001.

Circulating serum levels of cytokines and chemokines were measured within groups. The levels of CXCL-10 (IP-10) were significantly higher at day 4 post infection (48 hours after the first TLR administration) in the TLR group compared to the Control group (1050 pg/ml ±183.3 SEM vs. 430.6 pg/ml ±93.85 SEM, *p*<0.043) (Fig. 3A), suggesting that only the TLR agonists, but not DENV or DENV/TLR, induced the secretion of this chemokine in significant amounts compared to the Control group. Seventy-two hours after the secondary TLR stimulation (day 10), serum levels of CXCL-10 remained higher than those of the Control group, yet not statistically significant (data not shown).

Serum levels of IL1-Ra experienced a surge on day 4 after the infection in all groups that received the TLR agonists (Fig. 3B). Of interest, the levels of this cytokine were significantly higher in the TLR group when compared to the DENV or DENV/TLR groups (366.0 pg/ml ±38.41 SEM vs. 39.20 pg/ml ±5.725 SEM *p*<*0.008* and vs. 177.1 pg/ml ±37.15 SEM *p*<*0.012*, respectively). Meanwhile, the values of serum IL-1Ra in the DENV/TLR group were also significantly higher than in the DENV group (177.1 pg/ml ±37.15 SEM vs. 39.20 pg/ml ±5.725 SEM *p*<*0.063*). Serum levels of this cytokine continued to increase consistently by day 10, and remained elevated on day 15 post infection in the groups receiving TLRs (Fig. 3B).

We did not detect significant changes of any of the other 12 cytokines or chemokines tested, including IFN-α, IFN-γ, TNF-α and IL-12p40 among others (data not shown). Taken together, the data indicate that both CXC10 and IL-1Ra are major components of the inflammatory response induced by TLR activation but not by Dengue infection alone in rhesus macaques.

### 3.5. Effect of in vivo TLR stimulation on the humoral immune response to DENV

**Figure 4:**
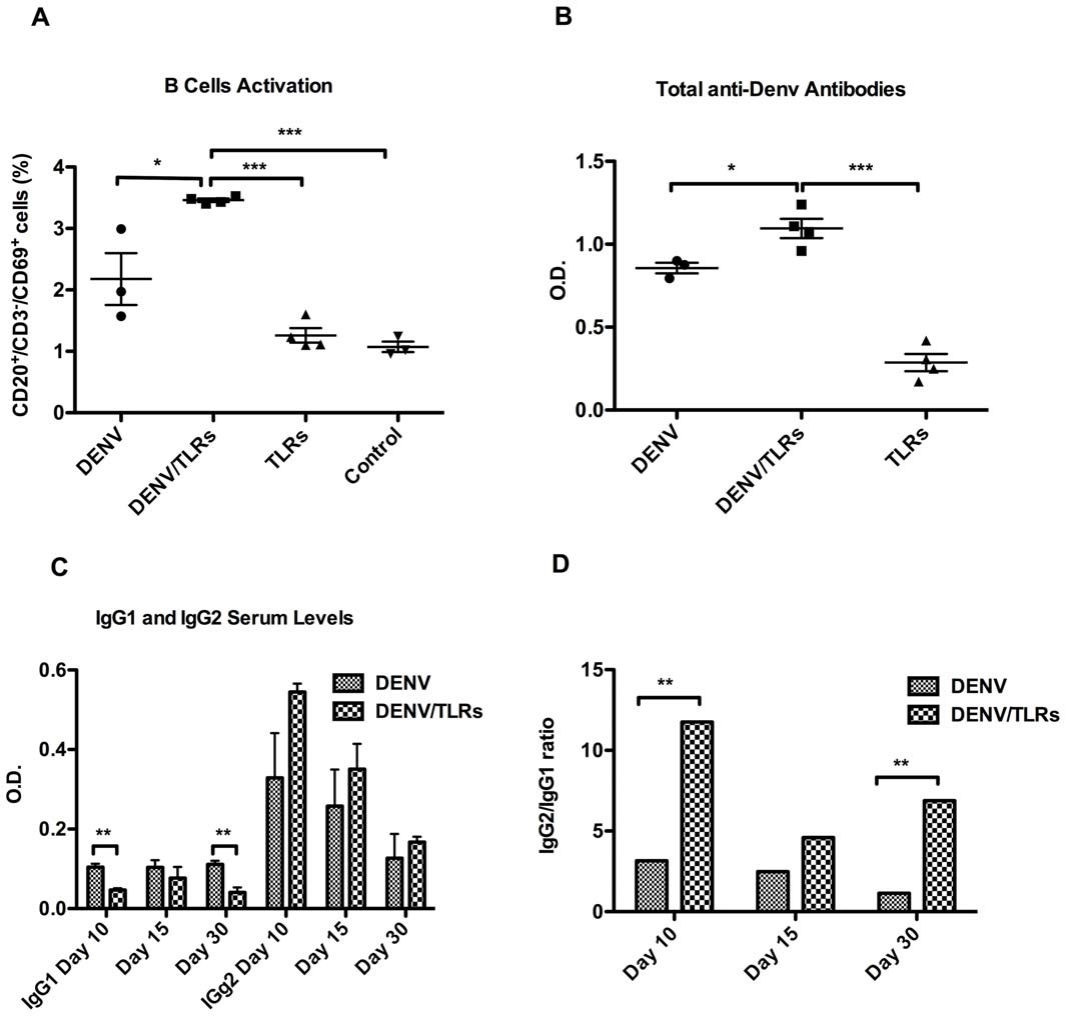
TLR3-7/8 stimulations in synergism with DENV induce quantitative and qualitative modifications of the humoral response. A: Ten days after the infection, the frequency of activated B cells was significantly higher in DENV/TLR group when compared to all other groups, showing a synergistic effect of DENV with the TLR agonists in this activation. DENV alone was able to induce activation of B cells when compared to the TLR and Control groups. However, in our model, TLR agonists alone were not sufficient to induce B-cell activation. B: The activation profile of B cells correlates with the levels of anti-DENV antibodies. Antibody levels were significantly higher in the DENV/TLR group compared to the DENV group. C: As early as 10 days after infection and coincident with activation of B cells, the switch to IgG1 was significantly diminished in the DENV/TLR group. This difference had disappeared by 15 days after the infection, but it was re-established on day 30. D: Although antibody O.D. values were limited, the difference in the IgG1 and IgG2 levels translated into a significant increase of the IgG2/IgG1 ratio (>11 and 6 times on days 10 and 30 after the infection, respectively). Asterisks denote significant differences, (*) p<0.05, (**) p<0.01, and (***) p<0.001.

Activated B cells, defined as CD20^+^/CD3^−^/CD69^+^, were detected on day 10 post infection (72 hours after secondary TLR stimulation) (Fig. 4A). Animals in the DENV/TLR group had the highest frequencies of activated B cells, and this difference reached statistical significance compared to the DENV group, (3.460% ±0.028% SEM vs. 2.177% ±0.422% SEM, *p*<*0.015*), the TLR group (3.460% ±0.028% SEM vs. 1.260% ±0.117% SEM, *p*<*0.060*), and the Control group (3.460% ±0.028% SEM vs. 1.073% ±0.085% SEM, *p*<*0.001*). Animals infected with DENV group did not differ in their frequencies of activated B cells from animals in the TLR group (2.177% ±0.422% SEM vs. 1.260% ±0.117% SEM *p = 0.06)*, or the Control group (1.073% ±0.085% SEM, *p = 0.06*) (Fig. 4A).

To test whether these differences in the frequencies of activated B cells translated into differences in antibody responses, we measured the level of total anti-DENV-specific antibodies in all groups. As shown in Fig. 4B, one month after the infection, DENV antibodies were significantly higher in the DENV/TLR group compared to the DENV group (*p*<*0.02*). We then tested whether TLR administration impacted antibody isotype switching. Serum samples were collected on days 10, 15 and 30 post infection from animals in the DENV/TLR and DENV groups and analyzed for DENV-specific IgG1 and IgG2 isotypes.

Surprisingly, and despite being still very low at the early time point of 10 days post infection, the levels of anti-DENV IgG1 were significantly lower in the DENV/TLRs group compared to DENV animals (*p*<*0.014)* (Fig. 4C). Significantly lower levels of IgG1 were also observed on day 30 post infection (*p*<*0.007*). A strong trend to higher values of IgG2 was observed in the DENV/TLR group, but did not reach statistical significance compared to the DENV group. However, this differential regulation of IgG isotypes resulted in a significantly higher IgG2/IgG1 ratio in the DENV/TLR group compared to the DENV group on days 10 (*p*<*0.001*) and 30 (p<*0.017*) after the infection (Fig. 4D).

Thirty days after the infection, neutralizing antibodies showed a trend to be higher in animals administered TLR/DENV compared to animals that were infected with DENV only (results no shown).

## Discussion

Dengue is an acute illness in which symptoms are present before full activation of the adaptive immune response takes place. The early recognition of virus infection by the innate immune system plays a critical role in priming and regulating the adaptive immune response. TLRs, which are especially abundant on effector cells of the innate immune response (macrophages, DC), are important in the initiation, shaping and regulation of the inflammatory response against viral diseases [39], [40]. Recently, it was shown that DENV differentially regulates several TLRs at the transcriptional level [6]. However, understanding the role of TLRs in the innate recognition and modulation of the adaptive immune response to DENV had not progressed due to the limitations of *in vivo* models for Dengue. We now provide evidence to support the hypothesis that maintenance of TLR-mediated responses, which are otherwise potentially countered by Dengue infection, may allow for greater control of viral replication.

Previously, it was shown that administration of multiple intravenous (i.v.) doses of the TLR3 agonist poly (ICLC) delayed the viremia in rhesus macaques infected with YF [30] and eliminated or delayed the viremia in animals challenged with VEE virus [31]. This effect on viremia was associated with the detection of IFN-α. Although, poly (I:C) is known to be a poor inducer of IFN-α in humans [41] and in non-human primates [33],[34], there are no available data on the impact of poly (I:C) on viremia in non-human primates and we did not identify a report on the effect of CL097M-012 (TLR-7/8 agonist) on any virus replication *in vivo*. However, our data are consistent with the YF and VEE reports, as *in vivo* administration of both TLR3 [poly (I:C)] and TLR-7/8 (CL097M-012) agonists at 48 hours after Dengue virus infection decreased viremia in 100% of the treated animals (Fig. 1B). To confirm the viremia results measured by qRT-PCR, we used the Platelia Dengue NS1 Ag Kit because it allowed us to measure NS1 protein in plasma samples and because of its high sensitivity (66%) and specificity (100%), as recently reported in tests of more than 800 samples from patients from Asia and Latin America [42]. In addition, this kit showed higher sensitivity (88%) in detecting DENV-1 than the other three DENV serotypes [42].

Induction of type-I IFN is expected to be associated with an anti-viral effect, although we were unable to detect type-I IFN in serum at any time point assayed after infection/stimulation (see below). A high clearance rate for circulating levels of type-I IFN may be associated with our failure to detect it, and future studies may need to collect samples closer to the time of TLR administration.

To delineate the contribution of innate effectors cells on the immune response, the frequencies of activated peripheral pDC and mDC were ascertained. Four days after DENV infection (48 hours after the first TLR administration), the highest level of mDC activation was observed in the TLR group whereas DENV infection alone did not result in activation of these cells (Fig. 2A, left panel), supporting the conclusion that TLR induction can overcome a virus-induced lack of innate activation responses in the presence of viremia.

Indeed, a negative effect of DENV infection on mDC activation was shown by the fact that animals in the DENV/TLR group had frequencies of activated mDC that were in between the values seen for animals in the DENV group and animals in the TLR group (Fig. 2A, left panel). This observed effect of DENV-mediated suppression of mDC activation is consistent with prior *in vitro* studies [23], [24], [43] and thus provides a link to *in vitro* effects within an *in vivo* model of infection. It is of interest to speculate whether this lack of mDC activation after infection may contribute to a decrease in adaptive immune responses. Notably, 10 days after the infection, animals in the DENV/TLR group showed higher frequencies of activated pDC when compared to the Control and DENV groups (Fig. 2B), suggesting that activation of DC is related to control of DENV (Fig. 1). Our results confirm previous finding showing that a blunted blood pDC response to dengue virus infection was associated with higher viremia levels, and with a pathogenetic cascade leading to severe disease [27].

Importantly, our *in vivo* data are consistent with previous *in vitro* findings demonstrating that DENV infection antagonizes the effect of the TLR-3 agonist poly (I:C) on DC activation (Fig. 2A, DENV/TLR vs. TLR group, *p*<*0.021*) [23], [44]. The mechanisms used by the virus to interfere with TLR signaling pathways require further investigation. However, we have evidence showing that DENV is able to block the activation and nuclear translocation of IRF3, a key player in the TLR3 pathway, induced by the agonists TLR3 poly (I:C) and LyoVec (Anglero, Y et al., unpublished results). The blockage of the activation of this regulatory factor by DENV after poly (I:C) treatment has been recently confirmed *in vitro* by others [44].

Furthermore, the inhibition of CXCL-10 secretion and of IL-1Ra by DENV is consistent with the profile of mDC deactivation, and emphasizes the potential role of DENV in suppressing TLR-induced inflammation. There are previous reports showing that poly (I:C) induces the activation of rhesus monkey DC *in vitro* [45] and that CXCL-10 levels in serum are mainly produced by DC after TLR-3 agonist stimulation [34]. Other *in vitro* studies have confirmed that DC produced CXCL-10 after DENV infection [21], [23], [46], but that its secretion was markedly reduced in DENV-infected cells when compared to non-infected bystander cells [21], [23]. Our present study shows concordance between TLR stimulation, DC activation (Fig. 2A left panel), CXCL-10 induction (Fig. 3A), and an immune response associated with control of DENV, whereas DENV alone is expected to act against these responses. In mouse models of DENV infection, it has been shown that CXCL-10 is required for resistance to primary DENV infection [47] by competitive inhibition of viral binding to heparan sulfate, the natural receptor of this virus.

The role of IL-1Ra in the pathogenesis of DENV is not fully understood. In one report, studying a cohort of 50 children, high levels of this cytokine were associated with high mortality [48], but in another report, high plasma levels of IL-1Ra were detected only in a limited proportion (16%) of adult patients showing mild symptoms [49]. Recently, transcriptional up-regulation of the IL-1Ra gene was associated with pleural effusion in a cohort of children with suspected diagnosis of DHF/DSS [6].

Compared to changes in IL1-Ra or CXCL-10, the pro-inflammatory cytokines are relatively short-lived and elevated concentrations are found only soon after the onset of serious infections [50], which could explain why we did not detect any of the other tested pro-inflammatory cytokines. Indeed, as our first sample collection was made 48 hours after the first TLR stimulation, we probably would have missed a transient peak in cytokine release that might have occurred before that time. Nevertheless, our results confirm a previous report in which no significant differences in serum levels of IFN-α, IFN-γ, TNF-α, IL12p40 and CCL3 were observed at 6, 24 or 48 hours between groups of rhesus macaques receiving the adjuvant KLH alone or together with poly (I:C) [34]. The absence of type-I IFN production after DENV infection *in vivo* reported in this work was first noticed by our group [28] and it has been confirmed recently *in vitro* in humans DC [44], [51].

Finally, we aimed to correlate differences in the innate responses observed between the various experimental groups to differences in DENV-specific immune responses. In recent years, there has been accumulating evidence that TLR agonists can directly activate B cells, promoting cell proliferation and IgG isotype switching [52], [53], [54]. In addition to B-cell activation, we also demonstrated a significant reduction of IgG1 concurrent with an increase of IgG2 antibodies (Fig. 4C). Our data are consistent with a report demonstrating that signaling through MyD88 (in our case stimulated by the TLR-7 agonist CL097M-012) may negatively regulate IgG1 and promote IgG2a/c antibodies [55].

The synergistic effect of TLRs with DENV infection on switching antibodies described in this work is of particular interest for DENV pathogenesis as it has been shown that IgG1 can fix complement more effectively than IgG2 [56], [57], thereby contributing to the development of DHF/DSS [58], [59]. The role of IgG1 in both the development and as a prognostic marker of severe clinical forms after DENV infection has been clearly demonstrated before [60]. Thus, TLR agonists can affect the type of antibody response that prevails after infection by eliciting a higher ratio of IgG2 to IgG1.

To our knowledge, this work presents the first data on the role of TLRs in the proliferation, activation and maturation of B-cell isotypes after DENV infection (or any other viral infection) in a higher animal model. Despite the limitation in the number of animals per group, our study provides definitive proof-of-concept data for how the innate response against DENV infection influence the molecular basis of the DENV–host interaction, from PRRs to the adaptive immune response, in an animal model, as well as insights into the mechanism by which DENV evades immune recognition and activation *in vivo*. Results from our work may have implications for the design of anti-DENV vaccine strategies through the potential use of TLR agonists as vaccine adjuvants or the exploration of activating TLR responses during active viremia as a strategy to modulate infection outcomes *in vivo*.
